# Two consecutive desensitisation protocols in a patient with anaphylaxis to colistin

**DOI:** 10.1186/2045-7022-4-S3-P62

**Published:** 2014-07-18

**Authors:** Maria Petrodimopoulou, Nikolaos Gavogiannakis, Vassileios Vovolis, Evagelia Kompoti

**Affiliations:** 1 Laikon General Hospital of Athens, Department of Allergology, Greece

## Introduction

To describe two distinct desensitisation protocols for colistin successfully used in the same patient in two different instances.

## Case summary

A 42-year old male suffering from chronic osteomyelytis of the left femoral bone due to Pseudomonas aeruginosa, was treated with intravenous colistin for 24 days. During this treatment he developed 3 mild reactions with rash and pruritus treated with anti-histamines. Eight months later he was readmitted in the hospital due to a relapse.Colistin was administered again as the pathogen was resistant to all other antibiotics. During the first intravenous infusion of colistin he developed severe anaphylaxis with generalised pruritus and erythema,low blood pressure, dyspnea, vommitting, diarrhea and feeling of imminent death. Skin prick and intradermal tests with the culprit drug were positive. A rush desensitisation protocol adapted from Tosi et al was implemented with only mild adverse reactions until therapeutic dose was reached. The patient tolerated colistin for 3 weeks. The drug was discontinued as new cultivations had to be obtained and was readministered 8 days later. Prior to the continuation of treatment new tests were performed with the SPT being negative this time, although intradermal tests were positive at the same dilution. Thus, a new faster and shorter protocol was developed and succefully tolerated by the patient without any reactions.

## Discussion

Increased incidence of serious infections caused by gram-negative bacteria led to greater use of colistin. Anaphylaxis to this antibiotic has rarely been reported. To the best of our knowledge this is the first case, in which an IgE mediated mechanism has been proven. We also demonstrate that tolerance after discontinuation of colistin, achieved by a desensitisation procedure, may last longer than it is generally believed for antibiotics.

## Conclusion

Colistin desensitisation can be a viable option for patients with serious gram-negative infections when no alternative drug is available.

